# Telerehabilitation in Physiotherapy Science: A Scoping Review

**DOI:** 10.7759/cureus.54396

**Published:** 2024-02-18

**Authors:** Magdalini Stamou, Christos Nikolaou, Savvas Chοiras

**Affiliations:** 1 Physiotherapy Department, University of West Attica, Athens, GRC

**Keywords:** internet-based rehabilitation, telephysiotherapy, e-health, telehealth, virtual reality rehabilitation, telerehabilitation, physical therapy, physiotherapy

## Abstract

The advancement of computer science technologies and telemedical devices has led to an increase in the use of telerehabilitation (TR) as a therapeutic intervention. In our days, TR interventions can be considered as alternative solutions to face-to-face therapy. The primary aim of this study is to evaluate whether TR can be effective in physiotherapy. This can be adjudicated by investigating the use of the TR applications, their cost effect, and the level of effectiveness each one of them can provide. Randomized controlled trials that were published between 2003 and 2023 in the English language and used TR as the intervention were collected from online databases (MEDLINE, Physiotherapy Evidence Database (PEDro), and Cochrane) to be reviewed. Twenty of them met the criteria and were included in the study. Studies meeting the inclusion criteria were categorized by the body system investigated. Out of the 20 studies that met the inclusive criteria, five are related to the musculoskeletal system, six are related to the nervous system, two are related to proprioception and balance, one is related to the respiratory system, one is related to the cardiovascular system, two are related to pelvic floor control, and three are related to autoimmune disorders. Studies have shown that implementing TR has resulted in significant improvements in terms of functionality, muscle strength, endurance, and self-improvement. Proprioception, autoimmune diseases, and cardiovascular health have shown the most improvement. The most commonly used tools for implementing TR are gamified virtual reality (VR) and digital apps. However, there are some disadvantages, such as the lack of personal contact and the cost involved. We found that TR has the potential to positively impact various health disorders, making it a suitable form of therapy for people who can't receive in-person treatment. Nonetheless, it cannot replace traditional physiotherapy, nor does it hold the same value as it.

## Introduction and background

The national physiotherapy associations are responsible for defining the roles of physiotherapy and physiotherapists relevant to their country's health service delivery needs, ensuring they are consistent with accepted international guidelines set out by World Physiotherapy. World Physiotherapy connects members, and regions, to high-quality knowledge for effective advocacy and evidence-based practice [1]. Physiotherapists provide services that develop, maintain, and restore people's movement and functional ability. As individuals age, suffer from injuries or diseases, or experience environmental factors that compromise their mobility and other functions, physiotherapists are equipped to assist them at any stage of life [2].

The field of rehabilitation holds significant importance in the professional world. Simply put, rehabilitation helps a child, adult, or an older person to be as independent as possible in daily activities and enables participation in education, work, and important life roles such as caring for the family. It does this, by working with the person and their family to address their underlying health conditions and symptoms, modifying their environment to better suit their needs, using assistive devices, and educating the person to enhance self-management and adapting tasks, so that they can perform more safely and independently. Rehabilitation can be provided in many places, such as hospital facilities, physical or occupational therapy centers, and the patient's environment [3].

Despite the benefits of rehabilitation and physiotherapy, these services are underutilized [4]. Due to low patient resources and high demand, services become saturated and waiting lists are created, limiting access [5,6].

Due to limited resources and evolving technology, where rehabilitation is necessary but not sufficiently implemented, alternative models have been created using new resources. Thus, telerehabilitation (TR) is proposed as a time- and resource-saving application in the healthcare system, aiming to improve accessibility for geographically remote populations with disabilities by implementing remote rehabilitation systems using telecommunications technologies [7,8]. The aim of digital technology services that can be offered with TR should be to provide a more accessible service to coordinate and securely transfer knowledge between professionals, care providers, and patients [9]. As part of the measures implemented because of the coronavirus disease 2019 (COVID-19) pandemic, TR is a process that allows continuity of care for patients who can benefit from remote consultations while ensuring greater protection for those in vulnerable groups [10-13]. This study aims to evaluate the effectiveness of TR in physiotherapy science, exploring the use of digital applications, the internet, and other devices in rehabilitation, as well as their cost-effectiveness.

## Review

Methods

**Figure 1:**
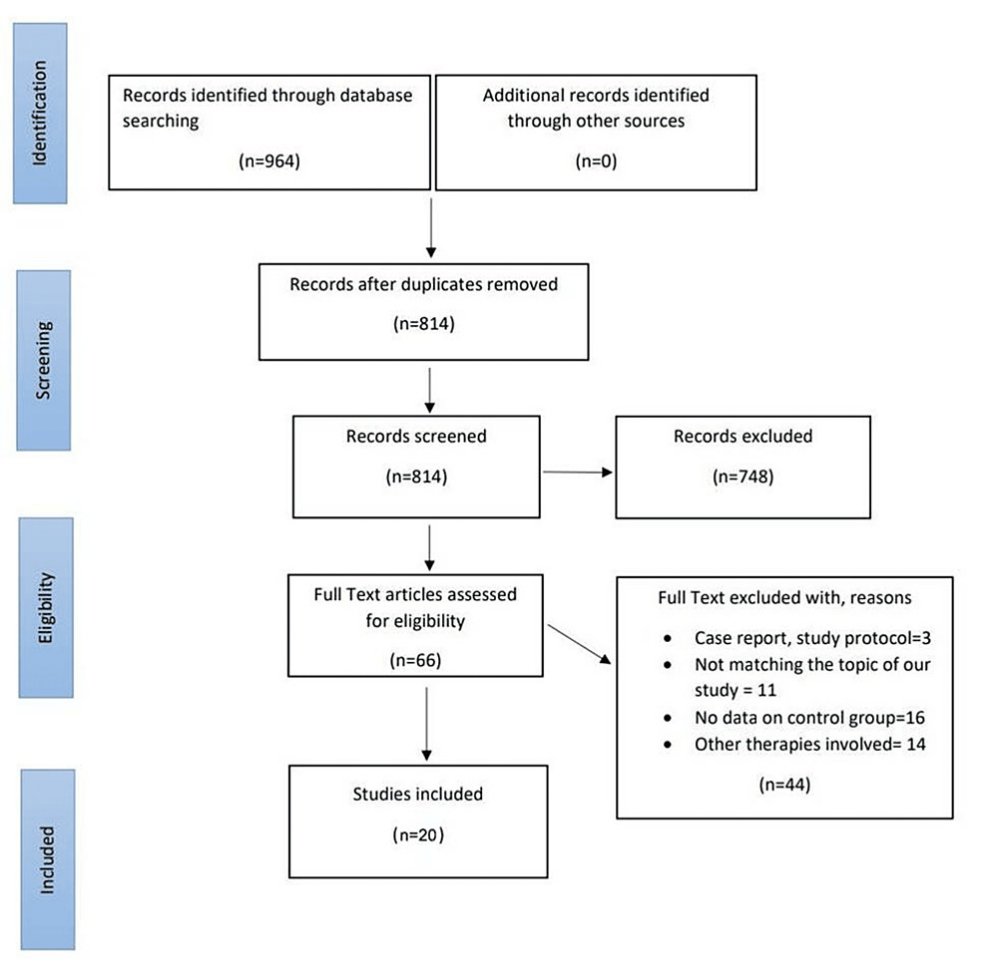
PRISMA flow diagram PRISMA: Preferred Reporting Items for Systematic Reviews and Meta-Analyses Reference: [14]

The scoping review was conducted following the Preferred Reporting Items for Systematic Reviews and Meta-Analyses (PRISMA) guidelines [14] (Figure 1) and registered on the Open Science Framework with identifier 10.17605/OSF.IO/KSC59.

Search Strategy 

In May 2023, a comprehensive literature search was conducted to find randomized controlled trials (RCTs) published over the last 20 years. The search was performed in the MEDLINE, Physiotherapy Evidence Database (PEDro), and Cochrane databases using Medical Subject Headings (MeSH) and free-text words related to "Internet-based rehabilitation and physiotherapy," "Telerehabilitation and physiotherapy," and "VR rehabilitation and physiotherapy." Only original articles in full text, written in English and published in peer-reviewed journals, were eligible for inclusion in the search. Initially, any articles that were duplicates were removed.

Risk of Bias

Following that, two reviewers (CN and SC) independently went through the titles, abstracts, and full text of the articles to see if they were eligible. Then, they examined the full text of the articles to make the final decision. If there was any disagreement, they discussed it and came to a consensus about whether to include the study or not. If needed, a third reviewer (MS) was consulted.

In this study, two authors (CN and SC) independently assessed the quality of the enrolled studies using the PEDro scale [15], which consists of 11 items that evaluate the external validity (item 1), internal validity (items 2-9), and statistical reporting (items 10-11). Detailed information about the PEDro scores can be found in Table 1.

**Table 1:** PEDro scale score [15] PEDro: Physiotherapy Evidence Database

	Rini et al. [16]	Gohir et al. [17]	Blanquero et al. [18]	Bäcker et al. [19]	Hodges et al. [20]	Galperin et al. [21]	Frevel et al. [22]	Feng et al. [23]	Gandolfi et al. [24]	Zak et al. [25]	Kayabinar et al. [26]	Ye et al. [27]	Sadeghi et al. [28]	Moy et al. [29]	Ögmundsdóttir Michelsen et al. [30]	Martinho et al. [31]	Wadensten et al. [32]	Rodríguez Sánchez-Laulhé et al. [33]	Galiano-Castillo et al. [34]	Dong et al. [35]
Eligibility criteria	+	+	+	-	+	+	+	+	+	-	+	+	+	+	+	+	+	+	+	+
Random allocation	+	+	+	+	+	+	+	+	+	+	+	+	+	+	+	+	+	+	+	+
Concealed allocation	+	+	+	+	-	+	+	-	-	-	-	+	-	-	+	+	+	+	+	+
Baseline similarity	+	+	+	+	+	+	+	+	+	+	+	+	+	+	+	+	+	+	+	+
Blinding of subjects	-	-	-	-	-	-	-	-	-	-	-	-	-	-	-	-	-	-	-	-
Blinding of therapist	-	-	+	-	-	-	-	-	-	-	-	-	-	-	-	-	-	-	-	-
Blinding of assessors	+	-	+	-	+	+	-	+	+	-	+	+	+	-	-	-	-	-	+	+
Adequate follow-up	+	-	+	-	-	+	+	+	+	+	-	+	+	+	-	-	+	-	+	-
Intention to treat	+	+	+	-	+	+	-	+	-	-	+	-	-	+	-	+	-	+	+	-
Between-group statistical comparisons	+	+	+	+	+	+	+	+	+	+	+	+	+	+	+	+	+	+	+	+
Point measures and measures of variability	+	+	+	-	+	+	+	+	+	+	+	+	-	+	+	+	+	+	+	+
Score	8/10	6/10	9/10	4/10	6/10	8/10	6/10	7/10	6/10	5/10	6/10	7/10	5/10	6/10	5/10	6/10	7/10	6/10	8/10	6/10

Results

The 20 studies that were included were about diseases related to the musculoskeletal system (five) [16,17,18,19,20], the nervous system (six) [20,21,22,21,24,25,26], the proprioception-balance system (two) [27,28], the respiratory system (one) [29], the cardiovascular system (one) [30], the pelvic floor control (two) [31,32], and the autoimmune diseases (three) [33,34,35].

Musculoskeletal System

Two studies [16,17] reported on participants who suffered from knee osteoarthritis. In the first study, the intervention group (IG) received a simulated pain management training program called Pain Coach. For the second study, a smartphone app was used as an intervention which provided daily range of motion exercises related to lower extremity neuromuscular strengthening and trunk stability. Both control groups (CGs) were given normal exercises. The first study reported significantly lower pain in the IG of women, and an increase in self-efficacy was demonstrated by both male and female subjects [16]. In the second study, the results showed a significant improvement in pain, stiffness, and physical function of the patients who were in the IG [17].

The studies [18,19] also found positive effects comparing the treatment group and CG. The first study used a digital app called ReHand for exercise and monitoring. The other study used an app called GenuSport, which was connected to a device, placed under the knee, to monitor the exercise as a game on a smartphone. In the study [20], the authors compared the use of a web-based intervention, MyBackPain, with unguided use of the internet for health education and advice related to back pain treatment. The results showed that unguided use of the MyBackPain platform had no positive effect. 

Nervous System

Out of six studies, two were about patients with multiple sclerosis [21,22], and two were about Parkinson's disease [23,24], while the remaining two articles referred to functional impairments due to old age and chronic stroke [25,26]. The studies [21,23,25,26] have used virtual reality (VR) devices for the IG. In the study [21], IG improved significantly in the Symbol Digit Modalities Test (SDMT), a commonly used test to assess psychomotor speed, compared to the CG. Contrary to the study [22] which used an internet-based home training program (eTraining) for balance exercises in the IG and hippotherapy in the CG, no statistically significant difference was observed. The study [23] found significantly better scores on the Berg Balance Scale (BBS), Timed Up and Go Test (TUG), and Functional Gait Assessment (FGA). The study [24] used a Tele Wii-Lab with a camera for the IG, resulting in improved mobility and dynamic balance for patients with caregivers. In the study [25], it was found that physiotherapy management when augmented by VR had a positive impact on individual functional performance. According to a study [26], chronic stroke patients underwent robot-assisted gait training (RAGT) using augmented VR. The IG received VR training, while the CG did not. The results showed an increase in walking speeds for participants in the IG, as well as an improvement in their cognitive performance. However, no such changes were observed in the CG. Nonetheless, there was no significant difference between the two groups in the post-treatment evaluations.

Proprioception and Balance

Two studies [27,28] conducted on elderly individuals (60 years or older) examined the relationship between proprioception and balance. [27] used an app that aimed at informing them about falls and providing informative videos. [28] used VR and balance training. The results of both studies showed a significant improvement in lower extremity strength and overall quality of life.

Respiratory System

The study [29] monitored the patients of the IG with a pedometer, and they were asked to provide their daily workouts to a website. The authors note that they didn't find any long-term effects after the intervention.

Cardiovascular System

From the sample, a study [30] was related to people with myocardial infarction. With their conventional cardiac rehabilitation, the IG had an extra treatment, the LifePod which was an app with information about a healthier lifestyle, risk factors, and possible symptoms of the condition. The study authors concluded that incorporating TR as an additional treatment led to a significantly greater reduction in blood pressure and there was a significant improvement in the score of the Healthy Diet Index.

Pelvic Floor Control

A study [31] was conducted to compare the effectiveness of VR and gym ball for strengthening the pelvic floor. However, the findings did not indicate any noteworthy variation between the two groups with statistical significance. Both interventions were found to be effective. Another study [32] focused on the urinary system. The intervention was carried out through a mobile phone application that offered pelvic floor muscle training, bladder training, and psychoeducation. Researchers concluded that the application was effective in improving urinary system health.

Autoimmune Diseases

The studies in this field [33,34,35] using cardiovascular exercises, self-management, and monitoring through the internet found improvements in gait, balance, muscle strength, and endurance and in the quality of life of the participants.

Discussion

In musculoskeletal-related conditions, it was observed that TR could cause an increase in participants' self-efficacy and functionality, while smaller positive effects were observed on pain-related anxiety. Furthermore, significant improvement in pain and flexibility was observed in almost all participants. As for the measurements, the data showed a positive effect on the Visual Analogue Scale for pain but also on the 10-m Walk Test. It's important to mention the reduction in taking painkillers, while at the same time, they were more likely to participate in a sport. On the other hand, one study found no significant findings [20]. In summary, TR seems to have improvements in patients' self-efficacy and walking ability.

People who participated in the neurological studies [21,22,23,24,25] presented positive results regarding the effect of each treatment. They showed extremely significant improvement in static and dynamic balance ability. Furthermore, TR induced a significant improvement in the SDMT [21]. Improvements were also observed in depressive symptoms, attention, and verbal fluency. It is important to mention the fact that TR helped the patients also clinically, as there were increases in the scores of the BBS, TUG, and FGA. In summary, all studies demonstrated significant improvements in posture, balance, and gait in both their static and dynamic forms.

Studies [27,28] that have analyzed the effect of TR on proprioception and balance have shown positive outcomes in IGs. Participants exhibited significant improvements in their overall condition, medication use, and lifestyle at home, as well as in leg strength, balance, and functional mobility.

In the only study [29] that was included based on respiratory conditions, the results according to the authors showed no significant difference between the IG and CG. They note that future studies should focus on long-term behavioral change in participants.

Another part where positive findings were found was in cardiovascular diseases, specifically in patients with myocardial infarction. The repeated measurements of the researchers showed a reduction in blood pressure, improvements in the quality of the diet, and a greater percentage compared to the CG who stopped smoking. They conclude that TR as an additional treatment can have positive results mainly in the first months after a myocardial infarction [30].

Regarding the studies of pelvic floor control [31,32], the results showed not only an overall improvement in muscle contraction but also a particularly significant improvement in incontinence symptoms. There was also an improvement in the ability to sustain pelvic floor muscle contraction, specifically an increase in endurance and average strength.

The study which referred to autoimmune diseases [33], specifically rheumatoid arthritis, concluded that the use of TR caused a significant improvement in the IG regarding the functionality of the patient's hand as well as a significant increase in performance at work. It is important to mention that there was a reduction in pain. The authors concluded that the treatment they studied is effective and recommend it for application in this population. Two studies [34,35] searching the effectiveness of TR in cancer patients had overall positive results. They report improvements in cognitive functions, hand symptoms, and overall pain levels. Positive changes were also found in the measurements of the Sit to Stand Test and Lift Arm Test, in the mental health, as well as in the quality of life of the patients.

The most positive effects caused by TR appeared in three categories: proprioception and balance disorders, autoimmune diseases, and diseases of the cardiovascular system.

In reflection of positive results, TR is also accompanied by many disadvantages, the biggest of which is the loss of face-to-face contact with the therapist. In second place comes the cost required to install and use any form of it. It is important to consider that the installation of special equipment is required, as well as familiarity with the specific method and technology in general. Furthermore, there is a possibility some therapies will be canceled due to technical problems.

Despite the different forms of TR, a particular form of intervention seems to dominate. Two tools were found to be the most effective interventions in aiding patients. The first was the use of VR, particularly through specially designed games. This method was particularly beneficial for patients with musculoskeletal and neurological conditions. The second tool involved the use of a specialized digital application, which was frequently used in studies related to musculoskeletal diseases. The application was typically accessed through a mobile phone.

## Conclusions

After reviewing the studies, it is apparent that TR offers a multitude of benefits across various populations and scenarios. Undoubtedly, it cannot replace in-person treatment, but it can be a viable option when that is not possible.

For future studies, we recommend extending the follow-up duration of patients to examine the long-term positive effects of therapy. In light of the foregoing, we recommend that prospective researchers undertake further consistency checks on exercise adherence and homework instructions. Such procedures are essential to ensure the credibility and validity of future studies. It is paramount that researchers proceed with utmost caution, diligence, and meticulousness in the execution of these procedures. By doing so, they will be able to establish a solid foundation for their research and contribute to the advancement of science.
